# Transient flow and heat transfer of CuO–Al_2_O_3_/H_2_O hybrid nanofluid flow over a radially stretching surface with dissipation and ohmic heating

**DOI:** 10.1186/s11671-026-04462-4

**Published:** 2026-03-18

**Authors:** M. Ragavi, T. Poornima, P. Sreenivasulu

**Affiliations:** 1 Department of Mathematics, School of Advanced Sciences, Vellore Institute of Technology, Vellore, 632014 India; 2 Department of Mathematics, School of Science and Humanities, Sri Balaji Chockalingam Engineering College, Arni, 632317 India; 3 Department of Mathematics, Sri Venkateswara College of Engineering, Tirupati, 517507 India

**Keywords:** Radial stretching sheet, Hybrid nanofluids, Viscous dissipation, Multiple linear regression, Keller box method, Ohmic heating

## Abstract

Incorporating suction, ohmic heating, and viscous dissipation, over a stretching sheet is crucial for accurately modeling thermal and fluid flow behavior in engineering and industrial applications. This study explores a hybrid nanofluid (CuO–Al_2_O_3_/H_2_O) transient thermal and flow characteristics as it flows over a radial stretching surface subjected to suction and viscous dissipation. The governing equations for momentum and energy are converted into a set of ordinary differential equations by the application of suitable similarity transformations. The Keller box technique solves the differential equations, resulting in a robust numerical solution. Additionally, multiple linear regression analysis is used to model the connections between the parameters statistically. The study reveals that suction and magnetic forces diminish the fluid flow profile, while the interplay between Eckert number and magnetic field intensity augments thermal distribution, leading to enhanced heat transfer efficiency. The tabular form illustrates the local variations in the friction factor and dimensionless temperature gradient based on different variables. The heat transfer behavior is notably affected by the Eckert number (0.1 ≤ E_C_ ≤ 0.7), magnetic parameter (0.2 ≤ M_g_ ≤ 0.8), unsteadiness parameter (0.1 ≤ A_P_ ≤ 0.4), and suction parameter (0.3 ≤ S_P_ ≤ 0.9). The findings of the current study were compared with earlier research, showing a high degree of correlation and supporting the validity of the present investigation. The multiple linear regression analysis shows substantial relationships between the parameters and the response variables, with high coefficients of determination (R^2^). This model demonstrates superior thermal performance, making it highly suitable for practical applications such as heat exchangers, cooling technologies, and electronic thermal management. The magnetic field facilitates precise manipulation of fluid flow, while the synergistic effects of viscous dissipation and suction enhance thermal management and stability, resulting in optimized system performance and reduced operational expenditures.

## Introduction

The ever-increasing demand for efficient heat transfer in various engineering applications, such as microelectronics cooling or energy conversion systems, has driven continuous research for innovative solutions. However, the emergence of nanofluids has significantly broadened the realm of improving heat transfer and fluid flow behaviors. In recent years, the idea of hybrid nanofluids has gained attention as a potential method to enhance heat transfer and fluid flow efficiency. The interaction between the hybrid nanofluid and the stretching sheet plays a crucial role in determining the fluid dynamics and heat transmission processes of the system. By understanding the fluid motion and thermal transfer mechanisms over-stretching surfaces, industries can optimize production processes, elevate product performance, optimize operations, reduce costs, and explore emerging technologies. Sakiadis [1] pioneered the study of flow over a stretching sheet, introducing the concept of a flat stretching sheet. Wang [2] examined the natural convection that occurs on a vertical, radially stretching surface. Zaimi et al. [3] looked into the steady flow of a nanofluid's boundary layer and the transfer of heat as it went over a sheet that was either stretching or shrinking with nonlinear permeability. In their investigation, they employed the shooting method.

The existence of a magnetic field leads to the generation of electromagnetic forces, which can influence and regulate the behavior of fluid flow and heat transfer processes. Sundar et al. [4] carried out the experimental determination of the convective heat transfer coefficient of MWCNT-Fe_3_O_4_–H_2_O hybrid nanofluid flowing through a circular tube. Anjali and Suriya [5] conducted a numerical study to examine the heat transfer rate on a permeable extendable surface using MHD hybrid nanofluid (Cu–Al_2_O_3_/H_2_O). Hayat et al. [6] studied the flow and thermal properties of a rotating hybrid nanofluid with partial slip and radiation. Waini et al. [7] characterized the heat transmission and movement of a hybrid nanofluid over a vertical thin needle. Dinarvand et al. [8] conducted a detailed analysis of the fluid flow properties of a hybrid nanofluid (CuO-Cu/blood) near a porous stretched surface under the influence of both external magnetic strength and convection. The results of their research have potential applications in biomedical fields, particularly in understanding the flow dynamics of the microcirculatory system and improving drug delivery methods. Tassaddiq et al. [9] examined the flow of an incompressible hybrid nanofluid over an impermeable rotating disk.

The ternary hybrid nanofluid improves heat transfer, and the nonlinear shrinking disk gives higher thermal performance than the linear case was analysed by Farah et al. [10]. Panda et al. [11] have reported the application of hybrid nanofluids in the design and optimization of heat-transfer systems that involve nonlinear radiation and hydromagnetic effects. Khan et al. [12] examined the hybrid nanofluids significantly improve heat transfer and surface friction over stretching and shrinking rotating disks, with the existence of dual solutions. Viharika et al. [13] studied the peristaltic flow of Phan–Thien–Tanner fluid with copper nanoparticles in an asymmetric channel, driven by the Lorentz force, focusing on entropy and biomedical applications. Their findings could be applied to fields such as polymer extrusion and metal spinning. Application of hybrid nanomaterials with enhanced thermophysical properties in solar-thermal heat exchangers was demonstrated by Khan et al. [14]. Salawu et al. [15] examined viscous heating in a hybrid nanofluid composed of multi-walled carbon nanotubes and iron oxide in water over a moving disk, using a non-uniform thermal model. Ismail and Gururaj [16] applied the shooting method to examine the magnetohydrodynamics movement of nanofluid with hybrid nanoparticles past a stretching cylinder embedded in spongy material, considering the influence of heat radiation. Application of Cu–Fe_3_O_4_/engine-oil Casson nanofluid for improving flow and thermal performance in parabolic trough solar collectors was investigated by Jamshed et al. [17]. Shahzad et al. [18] conducted a detailed analysis of hybrid nanofluid flow and heat transfer with entropy generation considerations in a parabolic trough surface collector of a solar-powered ship. Ragavi and Poornima [19] analysed the impact of nanoparticle shapes on the Ag–Al_2_O_3_/H_2_O hybrid nanofluid as it moves over an unsteady radially stretching sheet embedded in a porous medium, utilizing bvp4c solver.

Joule heating and viscous dissipation are crucial in determining the nanofluid's thermal properties and influencing its heat transfer behavior. Vinothkumar and Poornima [20] analysed the heat-transfer characteristics of a Jeffrey non-Newtonian fluid flowing over a vertical plate embedded in a non-Darcy porous medium. Shahzad et al. [21] performed a comprehensive analysis of the flow behavior of a non-Newtonian tangent hyperbolic nanofluid flowing past a stretching sheet, taking into account the effects of thermophoresis and Brownian motion. Yasir et al. [22] performed a numerical study on the magnetized flow characteristics of both MgO/H_2_O nanofluid and MgO–Ag/H_2_O hybrid nanofluid. Rasheed et al. [23] executed a numerical exploration to analyze the transient behavior of magnetohydrodynamic Jeffrey nanofluid flow over a stretchable vertical cylinder, considering the combined effects of mixed convection and thermally radiative transport. Yusuf et al. [24] studied the magnetohydrodynamic flow of a hybrid nanofluid around a rotating sphere, using methanol as the base fluid with AA7072 and AA7075 nanoparticles**.** Ragavi et al. [25] examined the axisymmetric flow behaviour of Ag–Gr/H_2_O hybrid nanofluid over a radially stretching surface, utilizing the Keller box method. Jothi and Lingaswamy [26] analysed water-based nanofluids containing BLF and CNFs to assess their heat and mass transfer performance over a rotating disk. Yasir et al. [27] investigated the Darcy–Forchheimer flow of hybrid nanofluids, consisting of ferrous ferric and zinc oxide nanoparticles suspended in a methanol-based base fluid, and analyzed the effects of rotation and velocity slip on the fluid’s behavior. Ontela et al. [28] analyzed the characteristics of a CuO–Cu hybrid nanofluid moving over a nonlinear stretching sheet in a porous medium, using response surface methodology to study its behavior.

Faraz et al. [29] investigated the effects of thermal radiation and mixed convection on axisymmetric Casson fluid flow in the presence of a magnetic field, employing the Keller Box method for their analysis. Khashi et al. [30] conducted a numerical study on the unsteady stagnation point flow of a hybrid nanofluid towards a radially shrinking Riga plate. Naseem et al. [31] examined the axisymmetric flow of a TiO2-water nanofluid over a porous surface that stretches radially, incorporating partial slip conditions at the boundary. Nihaal et al. [32] investigated heat and mass transfer in ternary nanofluid flow over a radially stretching sheet, utilizing the BVP4C method. Ali et al. [33] performed a numerical analysis of the thermal behavior of nanofluid and hybrid nanofluid flow over unsteady radially stretching surfaces.

A thorough literature review revealed that prior studies had not explored a hybrid nanofluid (CuO-Al_2_O_3_) flow and thermal behavior on a surface that extends radially using KBM and multiple linear regression. This article aims to examine and analyze nanofluid distinct flow and heat transfer characteristics over a surface to offer valuable insights for improving their thermal transport properties. Moreover, the model encompasses the impacts of key physical processes, including internal fluid friction, suction effects, and Joule heating, which makes the results more accurate. The combined effects of:

*Hybrid Fluid:* High intrinsic *k* (thermal conductivity).

*Suction:* Active removal of heat.

*Unsteadiness:* Passive confinement of the thermal field.

lead to the most significant increase in the Nusselt number, making this model the most efficient configuration for rapid cooling in manufacturing processes involving electrically conductive, moving surfaces.

By utilizing similarity transformation, the dimension equations are converted into non-dimensional equations for numerical operation. Next, we apply the Keller box method in MATLAB software to obtain the solutions. The study findings are presented using various graphs and tables. A multiple linear regression approach examines the relationships between the parameters, providing a statistical framework for understanding their interconnections and potential correlations. Understanding the velocity and thermal distribution over a surface is essential for optimizing its performance across various industrial sectors, including advanced thermal management systems, microfluidic devices, and thermal exchangers.

This study applies numerical and statistical methods to explore the below presented research questions.

1. What is the impact of the Eckert number, suction parameter, and magnetic parameter on the flow velocity and temperature profiles?
2. How do variations in the key governing parameters modulate the skin friction coefficient (surface shear stress) and Nusselt number (heat transfer rate)?
3. In what ways can multiple linear regression techniques be applied to analyze the relationships among the physical quantities under investigation systematically?
4. We expect that the overall synergy outcome as $Nu_{Hybrid,Transient} > Nu_{Mono,Steady}$

***Novelty****:* In this paper, we analyze the unsteady axisymmetric flow and thermal characteristics of a hybrid nanofluid (CuO–Al_2_O_3_) over a radially stretching sheet in the presence of suction, joule heating, and viscous dissipation. The Keller-box method combined with multiple linear regression is used to provide accurate results and simple predictive relations, an approach not applied in previous studies.

## Mathematical modelling

**Fig. 1:**
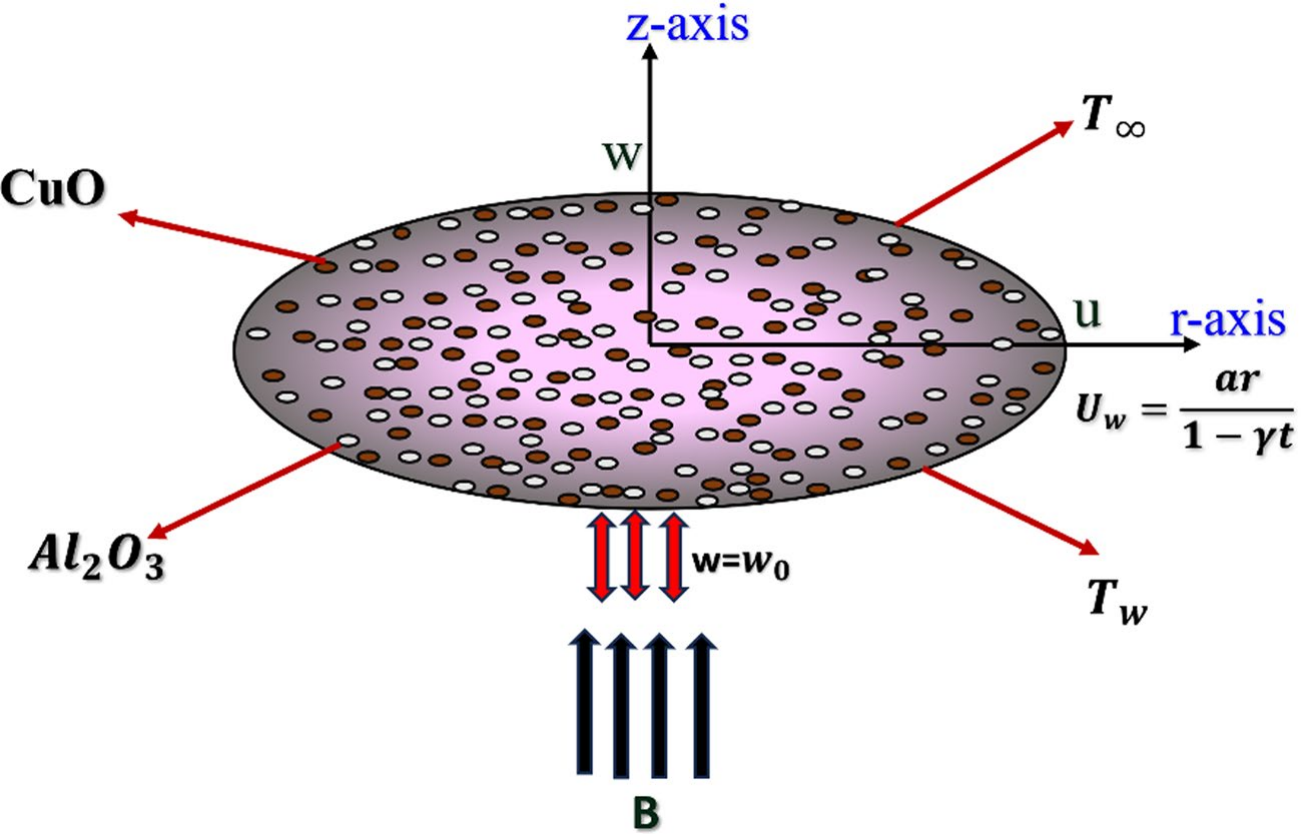
Schematic representation of the system

Assumptions of the study are as follows:

i. An unsteady, axisymmetric, 2D electrically conducting laminar flow of a hybrid nanofluid consisting of two types of nanoparticles (CuO-Al_2_O_3_) suspended in a base fluid (H_2_O) across a radially stretching surface. Figure 1 presents a physical model to facilitate analysis.
ii. The configuration consists of a sheet positioned at $z = 0$, which occupies the space $z > 0$.
iii. The fluid density is considered constant (incompressible), but the flow characteristics vary with time. There are no changes in the angular direction (axisymmetric).
iv. For computational purposes, a cylindrical polar coordinate system $(r, \theta , z)$ was adopted.
v. The sheet undergoes radial expansion with a velocity ${U}_{w}=\frac{ar}{1-\gamma t}$, the variables $a$ and $\gamma$ indicate parameters that describe the physical properties of the stretching surface.
vi. $T (r, z, t)$ represents the temperature, indicating its dependence on radial position $(r),$ axial position $(z),$ and time $(t).$ The velocity field $[v (r, z, t)]$ is a vector containing radial $(u)$ and axial $(w)$ components. The temperature of the fluid takes the form ${T}_{w}={T}_{\infty }+{T}_{r}\frac{a{r}^{2}}{2\nu {(1-\gamma t)}^{2}}.$ Here ${T}_{w}$ denotes the wall temperature, while ${T}_{\infty }$ denotes the far-field temperature.
vii. The flow described is axisymmetric, unsteady, incompressible, and laminar. A magnetic field of strength B $=\frac{{B}_{0}}{1-\gamma t}$, acts perpendicular to the stretching sheet.
viii. Suction occurs at the sheet, modeled by a prescribed wall-normal velocity component. Additionally, viscous dissipation is taken into account.
ix. An isothermal wall, which corresponds to the boundary condition $\mathrm{T}={\mathrm{T}}_{\mathrm{w}}$ at $\mathrm{z}=0$ was assumed.
x. The particles are assumed to be uniformly suspended and in thermal equilibrium with the base fluid.
xi. The nanoparticles are assumed to be spherical, uniform, and small enough (typically 100 nm so that the effects of agglomeration (clumping) and Brownian motion (random particle movement) on the macroscopic flow are either negligible or accounted for through *effective* thermal conductivity and viscosity models.
xii. It is assumed that the nanoparticles and the base fluid instantly reach the same temperature at any point. There is no thermal slip between the particles and the fluid.

According to the assumption mentioned above, we can represent the equation governing the current model as follows [29, 30]:

**Continuity equation:**

1 $$\frac{\partial u}{\partial r}+\frac{u}{r}+\frac{\partial w}{\partial z}=0$$

**Momentum equation:**

2 $$\frac{\partial u}{\partial t}+u\frac{\partial u}{\partial r}+w\frac{\partial u}{\partial z}=\frac{{\mu }_{hnf}}{{\rho }_{hnf}}\frac{{\partial }^{2}u}{\partial {z}^{2}}-\frac{{\sigma }_{hnf}{B}^{2}u}{{\rho }_{hnf}}$$

**Energy equation:**

3 $$\frac{\partial T}{\partial t}+u\frac{\partial T}{\partial r}+w\frac{\partial T}{\partial z}=\frac{{k}_{hnf}}{{\left(\rho {C}_{p}\right)}_{hnf}}\frac{{\partial }^{2}T}{\partial {z}^{2}}+\frac{{\mu }_{hnf}}{{\left(\rho {C}_{p}\right)}_{hnf}}{\left(\frac{\partial u}{\partial z}\right)}^{2}+\frac{{\sigma }_{hnf}{B}^{2}{u}^{2}}{{\left(\rho {C}_{p}\right)}_{hnf}}$$

The boundary conditions near and distant from the sheet are [29]:

4 $$\begin{gathered} u = U_{w} , w = w_{0} , T = T_{w} \quad \quad at\;z = 0 \hfill \\ u \to 0, T \to T_{\infty } \quad \quad at\;z \to \infty \hfill \\ \end{gathered} $$

Equation (4) describes the boundary conditions and it is very essential in fluid dynamics problems. The fluid tangential velocity at the wall equals the wall velocity. This is the no-slip condition, meaning the fluid sticks to the wall. The normal (vertical) velocity at the wall is prescribed. The mass transfer suction velocity is defined as ${w}_{0}=-2\sqrt{\frac{a{\nu }_{f}}{1-\gamma t}} S_{p}$ . This can represent suction or injection through the wall: $w_{0} < 0$: suction, $w_{0} > 0$: blowing/injection. The temperature at the wall is fixed at the wall temperature, meaning the wall is held at a constant thermal condition. Far away from the wall, the fluid velocity approaches zero, meaning the wall-driven flow dies out and the fluid is essentially undisturbed. Far from the wall, the temperature approaches the ambient free-stream temperature, indicating the thermal influence of the wall vanishes at large distance.

These boundary conditions specify the influence of the stretching surface on the fluid and ensure that the velocity and temperature profiles follow the correct physical behavior at the wall and in the far field.

The properties of hybrid nanofluid in terms of thermophysical characteristics are outlined as follows [30]:

$$\frac{{\mu }_{hnf}}{{\mu }_{f}}=\frac{1}{{\left(1-{\delta }_{1}\right)}^{2.5}{\left(1-{\delta }_{2}\right)}^{2.5}}$$

$${\rho }_{hnf}=\left(1-{\delta }_{2}\right)\left[\left(1-{\delta }_{1}\right){\rho }_{f}+{\delta }_{1}{\rho }_{s1}\right]+{\delta }_{2}{\rho }_{s2}$$

$${\left({\rho C}_{p}\right)}_{hnf}=\left(1-{\delta }_{2}\right)\left[\left(1-{\delta }_{1}\right){\left({\rho C}_{p}\right)}_{f}+{\delta }_{1}{\left({\rho C}_{p}\right)}_{s1}\right]+{\delta }_{2}{\left({\rho C}_{p}\right)}_{s2}$$

$$\frac{{k}_{hnf}}{{k}_{bf}}=\frac{{k}_{s2}+2{k}_{bf}-2{\delta }_{2}({k}_{bf}-{k}_{s2})}{{k}_{s2}+2{k}_{bf}+{\delta }_{2}({k}_{bf}-{k}_{s2})} , \frac{{k}_{bf}}{{k}_{f}}=\frac{{k}_{s1}+2{k}_{f}-2{\delta }_{1}({k}_{f}-{k}_{s1})}{{k}_{s1}+2{k}_{f}+{\delta }_{1}({k}_{f}-{k}_{s1})}$$

5 $$\frac{{\sigma }_{hnf}}{{\sigma }_{f}}=\left(1+\frac{3\left(\frac{{\sigma }_{s2}}{{\sigma }_{f}}-1\right){\delta }_{2}}{\left(\frac{{\sigma }_{s2}}{{\sigma }_{f}}+2\right)-\left(\frac{{\sigma }_{s2}}{{\sigma }_{f}}-1\right){\delta }_{2}} \times 1+\frac{3\left(\frac{{\sigma }_{s1}}{{\sigma }_{f}}-1\right){\delta }_{1}}{\left(\frac{{\sigma }_{s1}}{{\sigma }_{f}}+2\right)-\left(\frac{{\sigma }_{s1}}{{\sigma }_{f}}-1\right){\delta }_{1}}\right)$$

The variables ${\delta }_{1}$ and ${\delta }_{2}$ represent the volume fractions of CuO, and Al_2_O_3_ nanoparticles, respectively. In the aforementioned case, the expressions used are as follows: hnf stands for hybrid nanofluid, s_1_ represents the first solid particle (CuO), f denotes the working fluid, and the second solid particle (Al_2_O_3_) is denoted as s_2_. The thermophysical properties of the nanoparticles and base fluid are summarized in Table 1.

**Table 1:** Thermophysical attributes of base fluid and nanoparticles [34–38]

Physical characters	Al_2_O_3_	CuO	H_2_O
k (*W/mK*)	40	1.4013	0.613
*C*_*p*_ (*J/kgK*)	765	754	4179
*σ* (S/m)	$36.9\times$ 10^6^	$3.5\times$ 10^6^	5.5
*ρ* (*kg/m*^3^)	3970	2200	997.1
*µ*_*f*_ (*kg/(m.s)*)	–	–	8.9 × 10^–4^
*Pr*_*f*_	–	–	6.1

**Similarity transformations and dimensionless form:**

Our problem encompasses a system of interconnected PDEs. Consequently, scaling transformations are utilized to non-dimensionalize the governing equations.

The application of similarity reduction to the problem is illustrated below [29, 30]:

6 $$ \begin{gathered} \psi = \frac{{ - r^{2} \sqrt {a\nu_{f} } }}{{\sqrt {1 - \gamma t} }}f\left( \eta \right) \hfill \\ \eta = {\mathrm{z}}\sqrt {\frac{a}{{\nu_{f} \left( {1 - t\gamma } \right)}}} \hfill \\ \theta \left( \eta \right) = \frac{{T - T_{\infty } }}{{T_{r} \frac{{ar^{2} }}{{2\nu \left( {1 - \gamma t} \right)^{2} }}}} \hfill \\ \end{gathered} $$

The stream function (ψ) now represents both the longitudinal and transverse velocity components.

7 $$u=-\frac{1}{r}\frac{\partial \psi }{\partial z}={U}_{w}{f}^{\prime}\left(\eta \right), w=\frac{1}{r}\frac{\partial \psi }{\partial r}=-2\sqrt{\frac{a\vartheta }{1-\gamma t}}f\left(\eta \right).$$

This fulfills the continuity condition, as expressed in Eq. (1).

To facilitate analysis, similarity transformations are utilized to render the momentum and energy equations dimensionless.

8 $${{C}_{1}f}^{{\prime}{\prime}{\prime}}+2f{f}^{{\prime}{\prime}}-{\left({f}^{\prime}\right)}^{2}-{A}_{P}\left({f}^{\prime}+\frac{\eta }{2}{f}^{{\prime}{\prime}}\right)-{C}_{2}{M}_{g}{f}^{\prime}=0,$$

9 $$\frac{{{C}_{3}\theta }^{{\prime}{\prime}}}{Pr}+2f{\theta }^{\prime}-{2f}^{\prime}\theta +{C}_{4}{E}_{C}{\left({f}^{{\prime}{\prime}}\right)}^{2}-{A}_{P}\left(2\theta +{\frac{\eta }{2}\theta }^{\prime}\right)+{C}_{5}{M}_{g}{E}_{C}{\left({f}^{\prime}\right)}^{2}=0,$$

The boundary condition (4) now transforms into

10 $$ \begin{gathered} f\left( \eta \right) = S_{P} , f^{\prime } \left( \eta \right) = 1,\quad \quad \theta \left( \eta \right) = 1\;at\;\eta = 0, \hfill \\ f^{\prime } \left( \eta \right) \to 0, \theta \left( \eta \right) \to 0\quad {\text{as }}\eta \to \infty . \hfill \\ \end{gathered} $$

The variables C_1_, C_2_, C_3_, C_4_, and C_5_ in the aforementioned equation are explicitly defined as follows:

$${C}_{1}=\frac{\raisebox{1ex}{${\mu }_{hnf}$}\!\left/ \!\raisebox{-1ex}{${\mu }_{f}$}\right.}{\raisebox{1ex}{${\rho }_{hnf}$}\!\left/ \!\raisebox{-1ex}{${\rho }_{f}$}\right.},{C}_{2}=\frac{\raisebox{1ex}{${\sigma }_{hnf}$}\!\left/ \!\raisebox{-1ex}{${\sigma }_{f}$}\right.}{\raisebox{1ex}{${\rho }_{hnf}$}\!\left/ \!\raisebox{-1ex}{${\rho }_{f}$}\right.},{C}_{3}=\frac{\raisebox{1ex}{${k}_{hnf}$}\!\left/ \!\raisebox{-1ex}{${k}_{f}$}\right.}{\raisebox{1ex}{${\left({\rho C}_{p}\right)}_{hnf}$}\!\left/ \!\raisebox{-1ex}{${\left({\rho C}_{p}\right)}_{f}$}\right.},{C}_{4}=\frac{\raisebox{1ex}{${\mu }_{hnf}$}\!\left/ \!\raisebox{-1ex}{${\mu }_{f}$}\right.}{\raisebox{1ex}{${\left({\rho C}_{p}\right)}_{hnf}$}\!\left/ \!\raisebox{-1ex}{${\left({\rho C}_{p}\right)}_{f}$}\right.},{C}_{5}=\frac{\raisebox{1ex}{${\sigma }_{hnf}$}\!\left/ \!\raisebox{-1ex}{${\sigma }_{f}$}\right.}{\raisebox{1ex}{${\left({\rho C}_{p}\right)}_{hnf}$}\!\left/ \!\raisebox{-1ex}{${\left({\rho C}_{p}\right)}_{f}$}\right.}.$$

Here are some dimensionless constants:${A}_{P}=\frac{\gamma }{a};$ ${E}_{C}=\frac{a{\nu }_{f}}{{C}_{p}{T}_{r}} ;$ $Pr=\frac{\rho {C}_{p}{\nu }_{f}}{{k}_{f}}$; ${M}_{g}=\frac{{B}_{0}^{2}{\sigma }_{f}}{a{\rho }_{f}}$; $Re=\frac{r{U}_{w}}{{\nu }_{f}}$; ${S}_{P}=\frac{-{w}_{0}}{2}\sqrt{\frac{1-\gamma t}{a{\nu }_{f}}}>0$ represent suction.

## Physical quantities

The flow dynamics are primarily governed by the Nusselt number (*Nu*), and shear stress (*C*_*f*_), denoted as [39]

11 $$Nu=\frac{r{q}_{w}}{{K}_{f}\left({T}_{w}-{T}_{\infty }\right)}, {C}_{f}=\frac{{\tau }_{w}}{{\rho }_{f}{{U}_{w}}^{2}}.$$

where $${\tau }_{w}={\mu }_{hnf}\left[\frac{\partial u}{\partial z}\right], and \space {q}_{w}= -{k}_{hnf}\frac{\partial T}{\partial z}$$ at z = 0.

The values mentioned above correspond to the heat flux at the boundary and shear rate coefficient. Equation (11) can be simplified in non-dimensional form as follows:

12 $$ \begin{gathered} C_{f} \sqrt {Re} = \frac{{\mu_{hnf} }}{{\mu_{f} }}f^{\prime \prime } \left( 0 \right), \hfill \\ Re^{ - 1/2} Nu = - \frac{{k_{hnf} }}{{k_{f} }}\theta^{\prime } \left( 0 \right). \hfill \\ \end{gathered} $$

## Numerical procedure

To tackle the nonlinear ordinary differential equations (7) and (8) along with the boundary conditions (9), we implement a finite difference scheme based on the Keller box method. The primary stages required in this process include:

1. Transform the governing equations of the problem into a set of first-order ordinary differential equations.
2. Employ the central difference scheme to transform the system of first-order ordinary differential equations into a numerically solvable set of difference equations.
3. To solve the non-linear system arising from the discretization, the Newton-Quasi-linearization method is implemented.
4. The system of difference equations is tackled efficiently using a block matrix algorithm.

To express the problem as a first-order system, we require a new dependent variable (m, s, b).

13 $${f}^{\prime}=m, {m}^{\prime}=s, {\theta }^{\prime}=b.$$

Then,

14 $${C}_{1}{s}^{\prime} +2fs-{\left(m\right)}^{2}-{A}_{P}\left(m+\frac{\eta }{2}s\right)-m{C}_{2}{M}_{g}=0,$$

15 $${C}_{3}\frac{{b}^{\prime}}{Pr}+2fb-2m\theta +{{C}_{4}E}_{C}{\left(s\right)}^{2}- {A}_{P}\left(2\theta +\frac{\eta }{2}b\right)+{C}_{5}{M}_{g}{E}_{C}{m}^{2}=0,$$

the boundary condition transforms into

16 $$ \begin{gathered} f\left( \eta \right) = S_{P} , m\left( \eta \right) = 1, \theta \left( \eta \right) = 1\quad \quad at\;\eta = 0, \hfill \\ m\left( \eta \right) = 0, \theta \left( \eta \right) = 0\quad \quad as\;\eta \to \infty . \hfill \\ \end{gathered} $$

**i) Finite difference scheme:**

Equations (13–15) are subjected to the finite difference method, the outcome is the following system of equations:

17 $${f}_{j}-{f}_{j-1}-\frac{{h}_{j}}{2}\left({m}_{j}+{m}_{j-1}\right)=0.$$

18 $${m}_{j}-{m}_{j-1}-\frac{{h}_{j}}{2}\left({s}_{j}+{s}_{j-1}\right)=0.$$

19 $${\theta }_{j}-{\theta }_{j-1}-\frac{{h}_{j}}{2}\left({b}_{j}+{b}_{j-1}\right)=0.$$

20 $$ \begin{gathered} C_{1} \left( {s_{j} - s_{j - 1} } \right) + 2\frac{{h_{j} }}{2}\left( {f_{j} + f_{j - 1} } \right)\left( {s_{j} + s_{j - 1} } \right) - \frac{{h_{j} }}{2}\left( {m_{j} + m_{j - 1} } \right)^{2} \hfill \\ \quad - \frac{{h_{j} }}{2}A_{P} \left[ {\left( {m_{j} + m_{j - 1} } \right) + \frac{\eta }{2}\left( {s_{j} + s_{j - 1} } \right)} \right] - \frac{{h_{j} }}{2}\left( {m_{j} + m_{j - 1} } \right)\left( {C_{2} M_{g} } \right) = 0. \hfill \\ \end{gathered} $$

21 $$ \begin{gathered} \frac{{C_{3} }}{Pr}\left[ {b_{j} - b_{j - 1} } \right] + 2\frac{{h_{j} }}{2}\left( {f_{j} + f_{j - 1} } \right)\left( {b_{j} + b_{j - 1} } \right) - 2\frac{{h_{j} }}{2}\left( {m_{j} + m_{j - 1} } \right)\left( {\theta_{j} + \theta_{j - 1} } \right) \hfill \\ \quad + C_{4} E_{C} \frac{hj}{2}\left( {s_{j} + s_{j - 1} } \right)^{2} - A_{P} \frac{{h_{j} }}{2}\left[ {2(\theta_{j} + \theta_{j - 1} ) + \frac{\eta }{2}(b_{j} + b_{j - 1} )} \right] = 0. \hfill \\ \end{gathered} $$

The aforementioned equations hold for *j* = 1, 2, 3, … − 1, while the boundary conditions apply at *j* = 0 and *j* = *J* are

22 $$ \begin{gathered} f_{0} = S_{P} , m_{0} = 1, \theta_{0} = 1, \hfill \\ m_{J} = 0, \theta_{J} = 0. \hfill \\ \end{gathered} $$

**ii) Linearization**

Newton's method is employed because the transformed boundary layer equations are a system of non-linear Ordinary Differential Equations (ODEs). Applying the Keller box method (a finite difference scheme) to these non-linear equations results in a non-linear algebraic system at each spatial step. Newton's method is the solution to handle this non-linearity efficiently. Here are the major advantages:

- Assurance of convergence and accuracy
- Efficiency and computational
- Speed stability and robustness
- Direct solution of the boundary condition problems

The primary advantage of coupling Newton's method with the Keller Box discretization is transforming a complex, non-linear problem into a solvable linear system, which can be solved rapidly using standard techniques.

We employed Newton's method to determine the solution of the nonlinear Eqs. (17–21). Then, the subsequent $\left[{f}_{j}^{i}, {m}_{j}^{i}, { s}_{j}^{i}, {\theta }_{j}^{i}, { b}_{j}^{i}\right]$

 are introduced where *i* = 0, 1, 2 …, The subsequent iterations yielded the following results:

23 $$ \begin{gathered} f_{j}^{i + 1} = f_{j}^{i} + \delta f_{j}^{i} , m_{j}^{i + 1} = m_{j}^{i} + \delta m_{j}^{i} , s_{j}^{i + 1} = s_{j}^{i} + \delta s_{j}^{i} , \hfill \\ \theta_{j}^{i + 1} = \theta_{j}^{i} + \delta \theta_{j}^{i} , b_{j}^{i + 1} = b_{j}^{i} + \delta b_{j}^{i} \hfill \\ \end{gathered} $$

We then substituted the RHS (Right-Hand Side) expressions from Eqns. (17–21) and ignored higher-order terms involving δ. The linearized equations are presented below (to simplify notation, we have dropped the superscript i):

$$\delta {f}_{j}-\delta {f}_{j-1}-\frac{{h}_{j}}{2}\left(\delta {m}_{j}+{\delta m}_{j-1}\right)={\left({r}_{1}\right)}_{j,}$$

$$\delta {m}_{j}-\delta {m}_{j-1}-\frac{{h}_{j}}{2}\left(\delta {s}_{j}+{\delta s}_{j-1}\right)={\left({r}_{2}\right)}_{j},$$

24 $$\delta {\theta }_{j}-\delta {\theta }_{j-1}-\frac{{h}_{j}}{2}\left(\delta {b}_{j}+{\delta b}_{j-1}\right)={\left({r}_{3}\right)}_{j},$$

$${\left({a}_{1}\right)}_{j}\delta {s}_{j}+{\left({a}_{2}\right)}_{j}{\delta s}_{j-1}+{\left({a}_{3}\right)}_{j}\delta {m}_{j}+{\left({a}_{4}\right)}_{j}{\delta m}_{j-1}+{\left({a}_{5}\right)}_{j}\delta {f}_{j}+{\left({a}_{6}\right)}_{j}\delta {f}_{j-1}={\left({r}_{4}\right)}_{j},$$

$$ \begin{gathered} \left( {b_{1} } \right)_{j} \delta b_{j} + \left( {b_{2} } \right)_{j} \delta b_{{j - 1}} + \left( {b_{3} } \right)_{j} \delta \theta _{j} + \left( {b_{4} } \right)_{j} \delta \theta _{{j - 1}} + \left( {b_{5} } \right)_{j} \delta f_{j} + \left( {b_{6} } \right)_{j} \delta f_{{j - 1}} + \left( {b_{7} } \right)_{j} \delta m_{j} \hfill \\ \quad + \left( {b_{8} } \right)_{j} \delta m_{{j - 1}} + \left( {b_{9} } \right)_{j} \delta s_{j} + \left( {b_{{10}} } \right)_{j} \delta s_{{j - 1}} = \left( {r_{5} } \right)_{j} . \hfill \\ \end{gathered} $$

where

$${\left({a}_{1}\right)}_{j}={C}_{1}+{h}_{j}{f}_{j-\frac{1}{2}}-\frac{{h}_{j}}{2}\frac{\eta }{2}{A}_{P},$$

$${\left({a}_{2}\right)}_{j}={\left({a}_{1}\right)}_{j}-{2C}_{1},$$

$${\left({a}_{3}\right)}_{j}=-\frac{{h}_{j}}{2}\left({{C}_{2}M}_{g}\right){-h}_{j}{m}_{j-\frac{1}{2}}-\frac{{h}_{j}}{2}{A}_{P},{\left({a}_{4}\right)}_{j}={\left({a}_{3}\right)}_{j},$$

$${\left({a}_{5}\right)}_{j}=-{h}_{j}{s}_{j-\frac{1}{2}},{\left({a}_{6}\right)}_{j}={\left({a}_{5}\right)}_{j,}$$

$${\left({b}_{1}\right)}_{j}=\frac{{C}_{3}}{Pr}+\frac{2{h}_{j}}{2}{f}_{j-\frac{1}{2}}-\frac{{h}_{j}}{2}\frac{\eta }{2}{A}_{P},{\left({b}_{2}\right)}_{j}=-\frac{{C}_{3}}{Pr}+\frac{2{h}_{j}}{2}{f}_{j-\frac{1}{2}}-\frac{{h}_{j}}{2}\frac{\eta }{2}{A}_{P},$$

$${\left({b}_{3}\right)}_{j}=-{2A}_{P}\frac{{h}_{j}}{2}-2\frac{{h}_{j}}{4}{m}_{j-\frac{1}{2}},{\left({b}_{4}\right)}_{j}={\left({b}_{3}\right)}_{j},$$

$${\left({b}_{5}\right)}_{j}={-2\frac{{h}_{j}}{2}b}_{j-\frac{1}{2}},{\left({b}_{6}\right)}_{j}={\left({b}_{5}\right)}_{j},$$

$${\left({b}_{7}\right)}_{j}=-\frac{2{h}_{j}}{2}{\theta }_{j-\frac{1}{2}}+{C}_{5}{M}_{g}{E}_{C}{m}_{j-\frac{1}{2}}{h}_{j},{\left({b}_{8}\right)}_{j}={\left({b}_{7}\right)}_{j},$$

$${\left({b}_{9}\right)}_{j}={{C}_{4}E}_{C}{h}_{j}{s}_{j-\frac{1}{2}},{\left({b}_{10}\right)}_{j}={\left({b}_{9}\right)}_{j},$$

$${\left({r}_{1}\right)}_{j}={f}_{j-1}-{f}_{j}+{h}_{j}{m}_{j-\frac{1}{2}}, { \left({r}_{2}\right)}_{j}={m}_{j-1}-{m}_{j}+{h}_{j}{s}_{j-\frac{1}{2}}, {\left({r}_{3}\right)}_{j}={\theta }_{j-1}-{\theta }_{j}+{h}_{j}{b}_{j-\frac{1}{2}},$$

$${\left({r}_{4}\right)}_{j}={{C}_{1}(s}_{j-1}-{s}_{j})-2{h}_{j}{f}_{j-\frac{1}{2}}{s}_{j-\frac{1}{2}}+{h}_{j}{\left({m}_{j-\frac{1}{2}}\right)}^{2}+{A}_{P}{h}_{j}\left[{m}_{j-\frac{1}{2}}+\frac{\eta }{2}{s}_{j-\frac{1}{2}}\right]+{h}_{j}{m}_{j-\frac{1}{2}}\left({{C}_{2}M}_{g}\right),$$

$${\left({r}_{5}\right)}_{j}=\frac{{C}_{3}}{Pr}\left({b}_{j-1}-{b}_{j}\right)-2{h}_{j}{f}_{j-\frac{1}{2}}{b}_{j-\frac{1}{2}}+2{h}_{j}{m}_{j-\frac{1}{2}}{\theta }_{j-\frac{1}{2}}-{E}_{C}{C}_{4}{h}_{j}{\left({s}_{j-\frac{1}{2}}\right)}^{2}-{ E}_{C}{{M}_{g}C}_{5}{h}_{j}{\left({m}_{j-\frac{1}{2}}\right)}^{2}+{A}_{P}\left[{2\theta }_{j-\frac{1}{2}}+\frac{\eta }{2}{b}_{j-\frac{1}{2}}\right]{h}_{j.}$$

**iii) Block tridiagonal structure**:

The system of Eqs. (24) can be reformulated into a matrix notation as follows:

$$ \left[ {\begin{array}{*{20}c} {\begin{array}{*{20}c} {\left[ {A_{1} } \right]} & {\left[ {C_{1} } \right]} & {} & {} & {} & {} & {} & {} \\ {\left[ {B_{2} } \right]} & {\left[ {A_{2} } \right] } & { \left[ {C_{2} } \right]} & {} & {} & {} & {} & {} \\ {} & {\left[ {B_{3} } \right]} & {\left[ {A_{3} } \right] } & {\left[ {C_{3} } \right]} & {} & {} & {} & {} \\ {} & {} & {} & {} & \ddots & \ldots & {} & {} \\ {} & {} & {} & {} & {} & {\left[ {B_{j - 1} } \right]} & {[A_{j - 1} ]} & {\left[ {C_{j - 1} } \right]} \\ {} & {} & {} & {} & {} & {} & {\left[ {B_{j} } \right]} & {[A_{j} ]} \\ \end{array} } \\ \end{array} } \right]\left[ {\begin{array}{*{20}c} {\left[ {\delta_{1} } \right]} \\ {\left[ {\delta_{2} } \right]} \\ {\left[ {\delta_{3} } \right]} \\ \vdots \\ {\left[ {\delta_{j - 1} } \right]} \\ {\left[ {\delta_{J} } \right]} \\ \end{array} } \right] = \left[ {\begin{array}{*{20}c} {\left[ {r_{1} } \right]} \\ {\left[ {r_{2} } \right]} \\ {\left[ {r_{3} } \right]} \\ \vdots \\ {\left[ {r_{j - 1} } \right]} \\ {\left[ {r_{j} } \right]} \\ \end{array} } \right] $$

or

25 $$\left[A\right]\left[\delta \right]=[r]$$

26 $$\left[{A}_{1}\right]=\left[\begin{array}{ccccc}0& 0& 1& 0& 0\\ \raisebox{1ex}{$-{h}_{j}$}\!\left/ \!\raisebox{-1ex}{$2$}\right.& 0& 0& \raisebox{1ex}{$-{h}_{j}$}\!\left/ \!\raisebox{-1ex}{$2$}\right.& 0\\ 0& \raisebox{1ex}{$-{h}_{j}$}\!\left/ \!\raisebox{-1ex}{$2$}\right.& 0& 0& \raisebox{1ex}{$-{h}_{j}$}\!\left/ \!\raisebox{-1ex}{$2$}\right.\\ {\left({a}_{2}\right)}_{1}& 0& {\left({a}_{5}\right)}_{1}& {\left({a}_{1}\right)}_{1}& 0\\ {\left({b}_{10}\right)}_{1}& {\left({b}_{2}\right)}_{1}& {\left({b}_{5}\right)}_{1}& {\left({b}_{9}\right)}_{1}& {\left({b}_{1}\right)}_{1}\end{array}\right]$$

for $$2\le j\le J,$$

27 $$\left[{A}_{j}\right]=\left[\begin{array}{ccccc}\raisebox{1ex}{$-{h}_{j}$}\!\left/ \!\raisebox{-1ex}{$2$}\right.& 0& 1& 0& 0\\ -1& 0& 0& \raisebox{1ex}{$-{h}_{j}$}\!\left/ \!\raisebox{-1ex}{$2$}\right.& 0\\ 0& -1& 0& 0& \raisebox{1ex}{$-{h}_{j}$}\!\left/ \!\raisebox{-1ex}{$2$}\right.\\ {\left({a}_{4}\right)}_{j}& 0& {\left({a}_{5}\right)}_{j}& {\left({a}_{1}\right)}_{j}& 0\\ {\left({b}_{8}\right)}_{j}& {\left({b}_{4}\right)}_{j}& {\left({b}_{5}\right)}_{j}& {\left({b}_{9}\right)}_{j}& {\left({b}_{1}\right)}_{j}\end{array}\right]$$

28 $$\left[{B}_{j}\right]=\left[\begin{array}{ccccc}0& 0& -1& 0& 0\\ 0& 0& 0& \raisebox{1ex}{$-{h}_{j}$}\!\left/ \!\raisebox{-1ex}{$2$}\right.& 0\\ 0& 0& 0& 0& \raisebox{1ex}{$-{h}_{j}$}\!\left/ \!\raisebox{-1ex}{$2$}\right.\\ 0& 0& {\left({a}_{6}\right)}_{j}& {\left({a}_{2}\right)}_{j}& 0\\ 0& 0& {\left({b}_{6}\right)}_{j}& {\left({b}_{10}\right)}_{j}& {\left({b}_{2}\right)}_{j}\end{array}\right]$$

29 $$\left[{C}_{j}\right]=\left[\begin{array}{ccccc}-\raisebox{1ex}{${h}_{j}$}\!\left/ \!\raisebox{-1ex}{$2$}\right.& 0& 0& 0& 0\\ 1& 0& 0& 0& 0\\ 0& 1& 0& 0& 0\\ {\left({a}_{3}\right)}_{j}& 0& 0& 0& 0\\ {\left({b}_{7}\right)}_{j}& {\left({b}_{3}\right)}_{j}& 0& 0& 0\end{array}\right]$$

In the subsequent steps, we tackle Eq. (25) under the assumption of a non-singular matrix *A* and utilize LU decomposition to express it as:

30 $$\left[A\right]=\left[L\right]\left[U\right]$$

where

31 $$\left[L\right]=\left[\begin{array}{ccccc}{[\alpha }_{1}]& & & & \\ {[\beta }_{2}]& [{\alpha }_{2}]& & & \\ & & \ddots & & \\ & & & \left[{\alpha }_{J-1}\right]& \\ & & & \left[{\beta }_{J}\right]& \left[{\alpha }_{J}\right]\end{array}\right], \left[U\right]=\left[\begin{array}{ccccc}{[I}_{1}]& {[\gamma }_{1}]& & & \\ & {[I}_{2]}& {[\gamma }_{2}]& & \\ & & \ddots & & \\ & & & \left[{I}_{J-1}\right]& \left[{\gamma }_{J-1}\right]\\ & & & & \left[{I}_{J}\right]\end{array}\right]$$

Equation (25) is modified by inserting Eq. (31) into it,

32 $$\left[L\right]\left[U\right]\left[\delta \right]=\left[r\right].$$

Let

33 $$\left[U\right]\left[\delta \right]=\left[S\right].$$

Then,

34 $$\left[L\right]\left[S\right]=\left[r\right].$$

where

35 $$\left[S\right]=\left[\begin{array}{c}\left[{S}_{1}\right]\\ \left[{S}_{2}\right]\\ \vdots \\ \left[{S}_{J-1}\right]\\ \left[{S}_{J}\right]\end{array}\right]$$

In this context, [${S}_{j}$] takes the form of a 5 × 1 column matrix, with its elements determinable by solving Eq. (32), which consists of the following:

36 $$\left[{\alpha }_{1}\right]\left[{S}_{1}\right]=\left[{r}_{1}\right], \left[{\alpha }_{j}\right]\left[{S}_{j}\right]=\left[{r}_{j}-{ \beta }_{j}\right]\left[{S}_{j-1}\right], 2\le j\le J.$$

A forward sweep have been utilized to determine ${\gamma }_{j}$, ${\alpha }_{j}$, and ${S}_{j}$ values. Subsequently, Eq. (33) is employed in a backward sweep to calculate the components of δ, resulting in the following outcomes:

37 $$\left[{\delta }_{J}\right]=\left[{S}_{J}\right], \left[{\delta }_{j}\right]=\left[{S}_{j}\right]- \left[{\gamma }_{j}\right]\left[{\delta }_{j+1}\right], 1\le j\le J-1.$$

Iterations are performed repeatedly until the desired level of accuracy $\left|\delta {v}_{o}^{\left(i\right)}\right|<\epsilon$ is achieved.

## Results and discussion

This section explores the various important factors that influence the velocity and temperature distribution on the surface. The numerical results presented in the tables and figures illustrate the significant effects of different physical parameters including, suction (S_p_), magnetic parameter (M_g_), Eckert number (E_C_), and unsteadiness parameter (A_P_) on the flow and thermal variations. The physical parameters are held constant at the following values: Prandtl number (Pr = 6.2), suction parameter (S_P_ = 0.5), magnetic parameter (M_g_ = 0.2), unsteadiness parameter (A_P_ = 0.3), Eckert number (E_C_ = 0.2), and ${\delta }_{1}={\delta }_{2}$= 0.01, except for the variable factor demonstrated in the accompanying figures and data tables. Moreover, the computed values of the friction factor (${C}_{f}{Re}$^1/2^) and dimensionless heat transfer coefficient ($Nu{Re}$^-1/2^) are presented. Table 2 compares the current numerical results for $f^{\prime\prime}(0)$ with the previous findings of Saini et al. [40] demonstrating excellent agreement and confirming the reliability and precision of the present numerical results.

**Table 2:** Comparison with existing results (${M}_{g}{=\delta }_{1}={\delta }_{2}=0,and {A}_{P}=0.5$)

*S*_*P*_	[30]	Current result
− 1	− 0.620400	− 0.620408
− 0.5	− 0.887200	− 0.887201
1	− 2.655999	− 2.656000

**Fig. 2:**
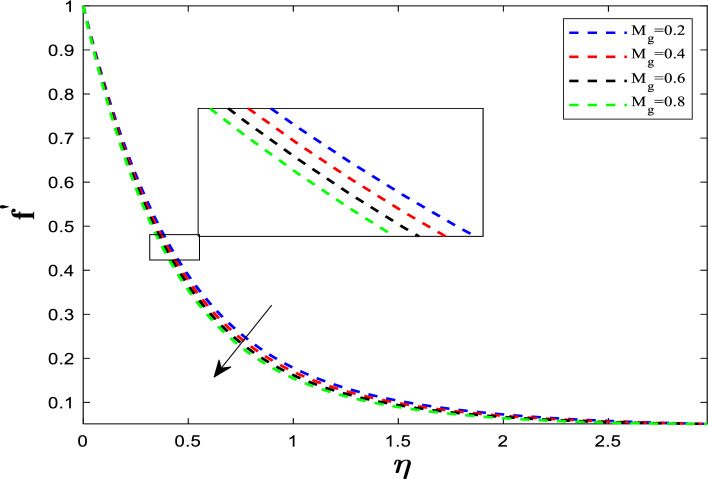
Influence of M_g_ on velocity distribution

The flow behavior of a fluid in the vicinity of a surface is entirely determined by its velocity distribution. Moreover, external forces like magnetic fields can significantly influence fluid motion, leading to changes in its flow patterns. Figure 2 depicts the connection between the velocity distribution and the magnetic field parameter (M_g_), the ratio of the electromagnetic body force (Lorentz force) to the viscous force in the fluid.

$$M_{g} = \frac{Magnetic\,\,body\,force}{{viscous\,force}}$$.

**Fig. 3:**
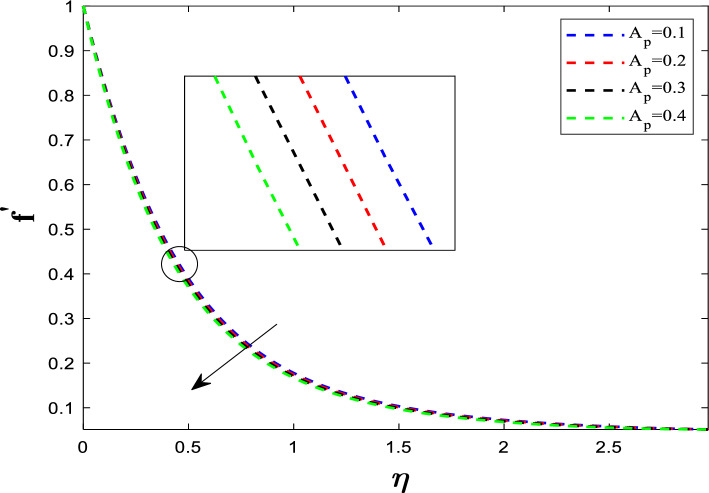
Influence of A_P_ on velocity distribution

When the electrically conductive hybrid nanofluid flows through the transverse magnetic field ($B$), an electric current is induced. This current interacts with the magnetic field to create the Lorentz force ($J \times B$), which opposes the fluid's velocity. Figure 3 illustrates the effect of the unsteadiness parameter (A_P_) on the flow profile. Unsteadiness is defined as:

$$ A_{p} \propto \frac{Rate\,of\,change\,in\,the\,system}{{Rate\,of\,viscous/thermal\,diffusion}} $$

**Fig. 4:**
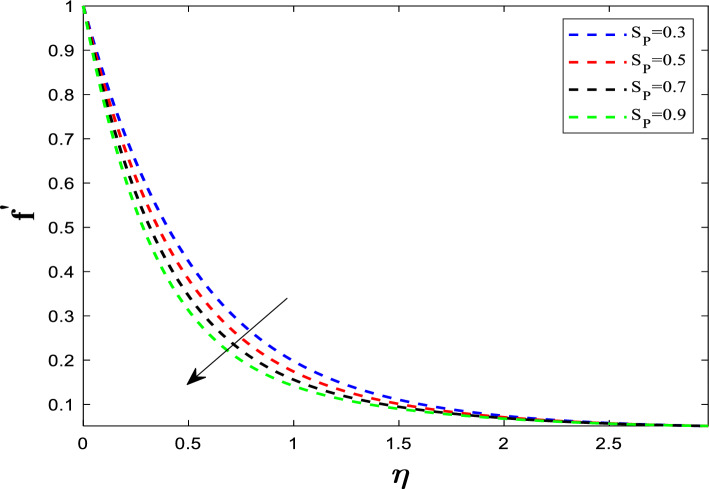
Influence of S_P_ on velocity distribution

When the unsteadiness parameter *A*_*p*_ is large, the fluid's inertia is high relative to the viscous forces. This high inertia resists the rapid changes in the stretching sheet's velocity. The fluid cannot accelerate as quickly to match the sheet's speed. Because the flow is decelerated near the wall, the velocity gradient becomes steeper. A steeper gradient at the wall means higher frictional resistance (drag). Figure 4 illustrates the effect of the suction parameter (S_P_) on ${f}^{\prime}\left(\eta \right)$. Increasing suction pulls the fluid toward the wall**.** The flow distribution undergoes a reduction as the suction parameter is elevated. The flow is confined or compressed closer to the wall. This leads to a steeper velocity gradient near the surface. The increased velocity gradient at the wall means the surface experiences higher drag (skin friction coefficient increases).

**Fig. 5:**
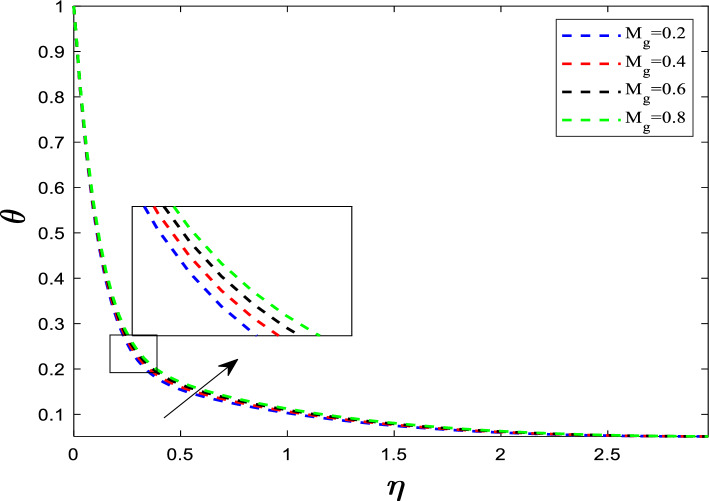
Influence of M_g_ on temperature distribution

**Fig. 6:**
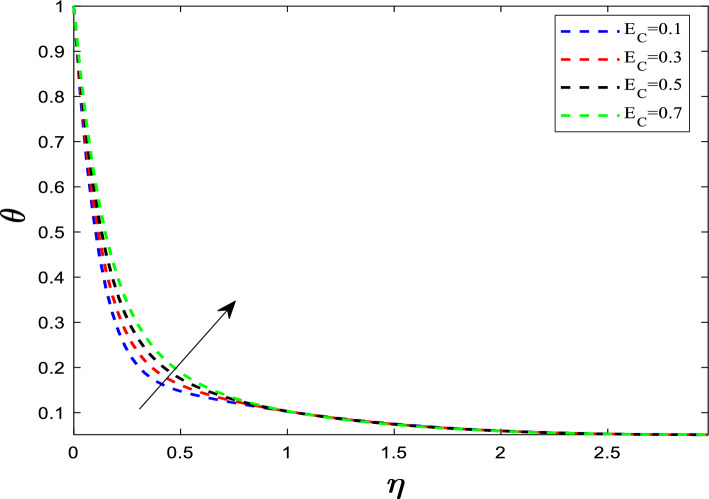
Influence of E_C_ on temperature distribution

As shown in Fig. 5, the temperature profile exhibits a consistent upward trend in response to increasing values of the magnetic parameter (M_g_). This relationship arises because the magnetic field induces a Lorentz force, which affects the motion of the fluid. The increased magnetic parameter enhances the resistance to fluid flow, decreases the fluid velocity means the fluid spends more time in the heated region near the stretching surface. This leads to increased heat absorption by the fluid. If the surface is hot, the temperature of the fluid layer increases, contributing to a thicker thermal boundary layer. The Eckert number quantifies the conversion of kinetic energy into thermal energy through viscous dissipation (Fig. 6).

$$ E_{C} \propto \frac{Heat\,generated\,by\,friction}{{Heat\,carried\,away\,by\,conduction/convection}} $$

**Fig. 7:**
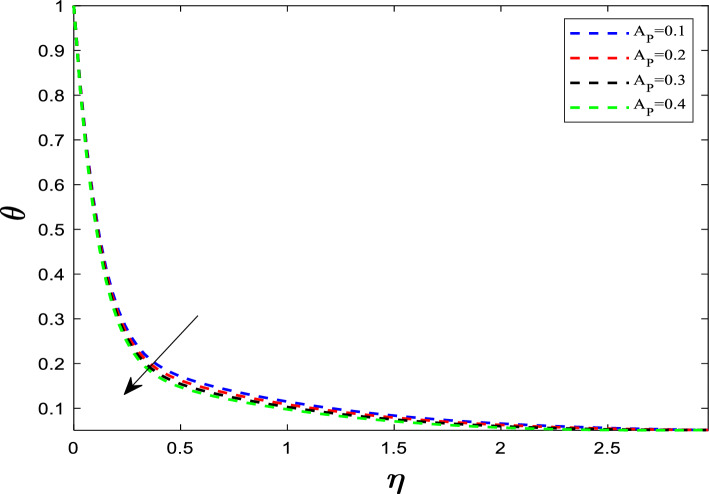
Influence of A_P_ on temperature distribution

A large Eckert number means that the flow velocity ${U}_{w}$ is high compared to the temperature difference $(\Delta T)$. This high velocity leads to high internal friction. The heat generated by this friction is trapped within the thermal boundary layer, which cannot be easily carried away by the fluid's specific heat capacity. This rises the temperature profile. Figure 7 elucidates the influence of the unsteadiness parameter (A_p_) on the temperature distribution. Since the flow is transient (changing rapidly), the heat does not have enough time to diffuse fully into the fluid. The rapid nature of the flow *sweeps* the heat away from the boundary more quickly (convective cooling dominates diffusive heating). As the unsteadiness parameter increases, the temperature profile exhibits a downward trend. Because the temperature near the wall drops quickly, the temperature gradient at the wall becomes steeper. A steeper temperature gradient means the heat is being transferred out of the sheet faster. In particular unsteadiness parameter acts as a control switch in confining the boundary layer.

**Fig. 8:**
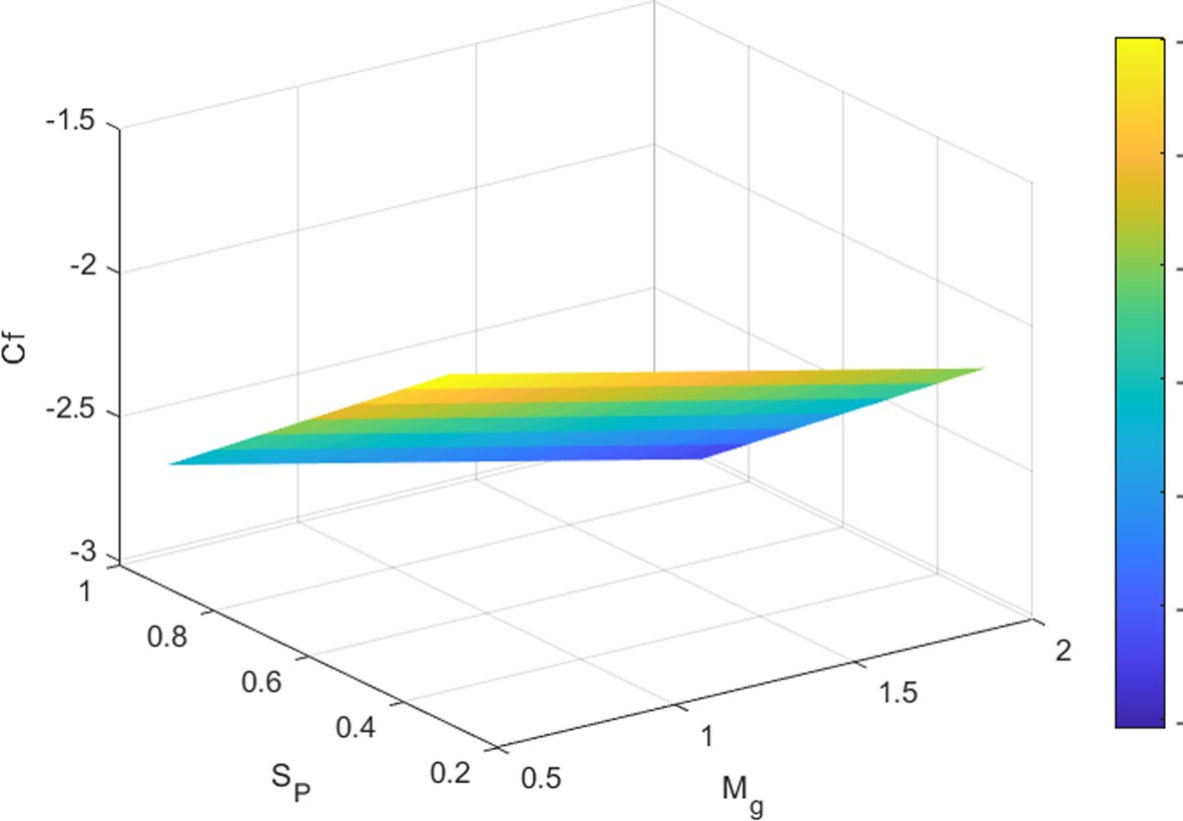
Influences of M_g_ and S_P_ on skin friction

**Fig. 9:**
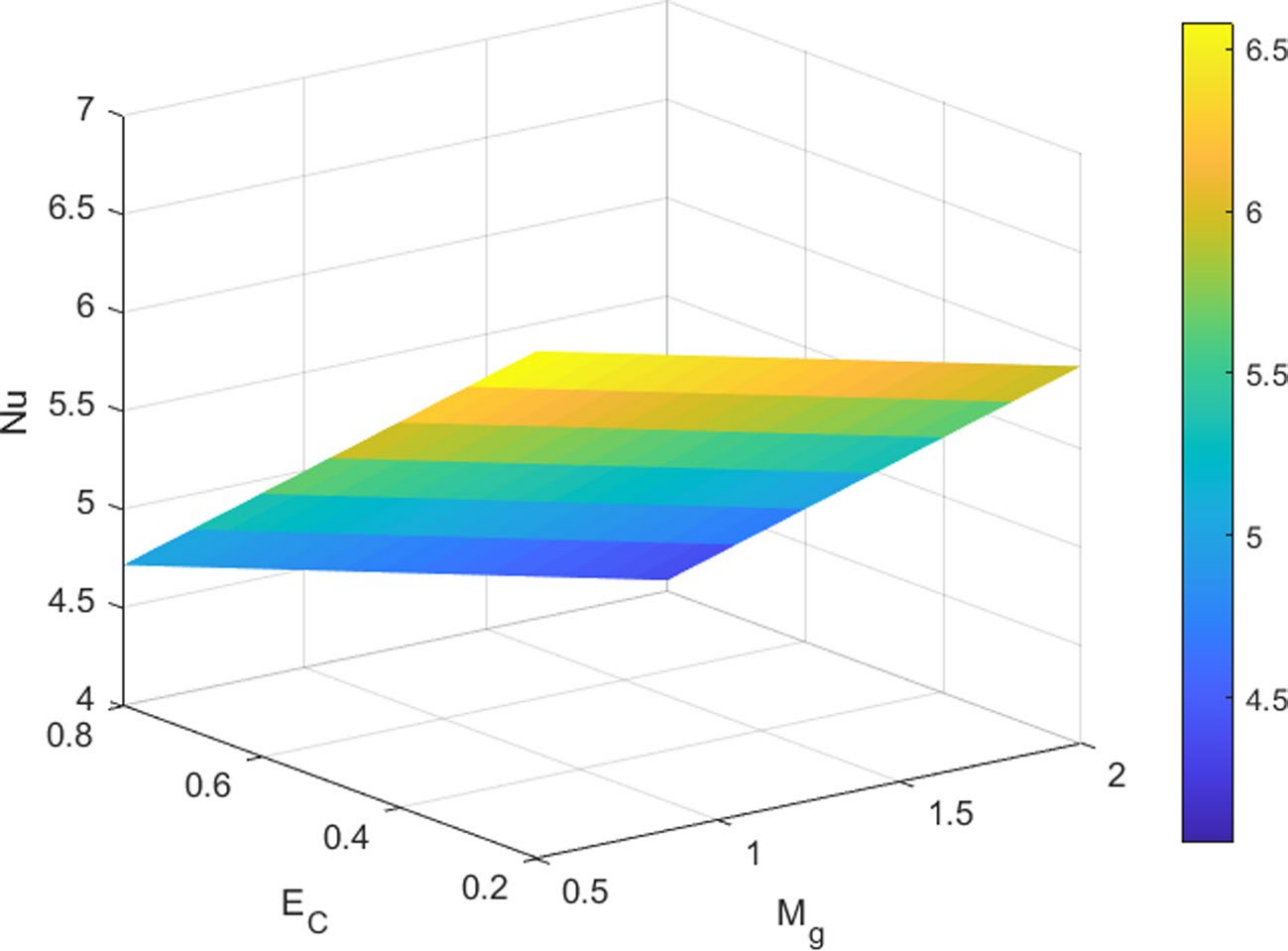
Influences of M_g_ and E_C_ on Nusselt number

**Fig. 10:**
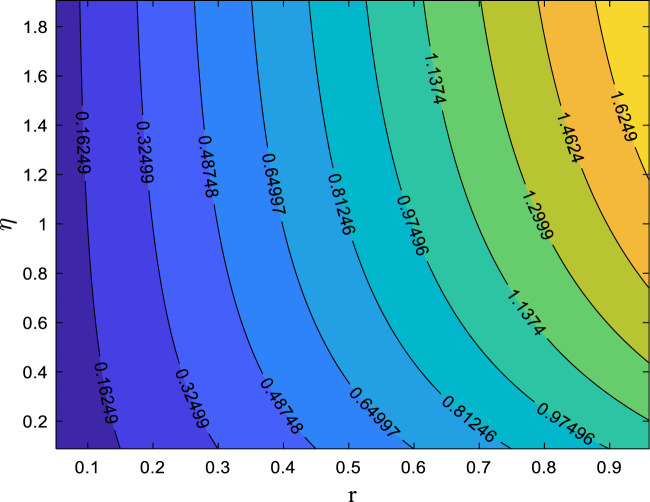
Streamline pattern when S_P_ = 0.5

**Fig. 11:**
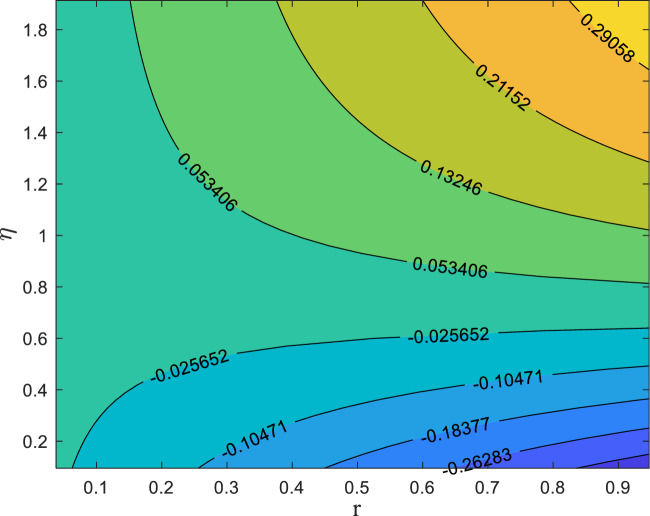
Streamline pattern when S_P_ = − 0.5

Tables 3 and 4 quantify the effects of suction, unsteadiness, Eckert number, and magnetic field on skin friction coefficient and thermal transmission rate. The analysis reveals a reduction in friction factor as the unsteadiness parameter (A_P_), suction parameter (S_P_), and magnetic parameter (M_g_) increase. An analysis of Table 4 suggests that increasing the magnetic parameter (M_g_) and unsteadiness parameter (A_p_) results in an enhancement of the dimensionless heat transfer coefficient, indicating enhanced thermal transport. Conversely, the Eckert number (E_C_) shows a decreasing trend. The 3D surface plots in Figs. 8 and 9 provide a visual representation of the influence of multiple factors on $${C}_{f}{Re}^{\raisebox{1ex}{$1$}\!\left/ \!\raisebox{-1ex}{$2$}\right.}$$(friction factor) and $${\mathrm{NuRe}}^{\raisebox{1ex}{$-1$}\!\left/ \!\raisebox{-1ex}{$2$}\right.}$$ (Nusselt number). Figure 8 demonstrates a clear reduction in $$\text{skin friction coefficient}$$ with increasing magnetic field strength and suction, while Fig. 9 shows a corresponding decrease in Nusselt number as magnetic field strength and Eckert number increase. Figures 10 and 11 present the streamline pattern. Streamlines illustrate the flow path and clarify how the velocity field evolves along the radially stretching surface, supporting the physical interpretation of the numerical results. It is noted that streamlines of suction sucks the fluid and particles towards the sheet and injection streamlines blows them away from the sheet. Suction (*S*_*p*_ > 0) extracts fluid from the surface, thinning the boundary layer and stabilizing the flow, which results in denser streamlines near the surface. The effect is to confine or compress the boundary layer. The streamlines (which represent the path of fluid particles) are indeed denser near the surface because the flow field is being pulled closer to the sheet, resulting in a steeper velocity gradient and higher shear stress. In contrast, injection (*S*_*p*_ < 0) introduces fluid into the boundary layer, thickening it and creating a more diffused streamline pattern. This visualization tool is essential for engineers and researchers can gain a deeper understanding of flow patterns. The streamlines are more diffused because the injection of fluid pushes the entire velocity field further away from the surface, causing the boundary layer to thicken. The velocity gradient at the wall becomes shallower, resulting in a decrease in shear stress (lower skin friction).

**Table 3:** Numerical data for skin friction coefficient when ${\delta }_{1}={\delta }_{2}$= *0.01*

$${M}_{g}$$	$${S}_{p}$$	$${A}_{p}$$	$${C}_{f}{Re}^{\raisebox{1ex}{$1$}\!\left/ \!\raisebox{-1ex}{$2$}\right.}$$
0.2			− 1.942717
0.4	0.5		− 2.011074
0.6			− 2.076747
0.8		0.3	− 2.139997
	0.3		− 1.729397
	0.5		− 1.977249
	0.7		− 2.242602
0.2	0.9		− 2.523399
		0.1	− 1.890415
	0.5	0.2	− 1.916732
		0.3	− 1.942717
		0.4	− 1.968377

**Table 4:** Numerical Data for Nusselt number when ${\delta }_{1}={\delta }_{2}$= *0.01*

${E}_{C}$	${M}_{g}$	${A}_{p}$	$Nu{Re}^{\frac{-1}{2}}$
0.1			7.549365
0.3	0.2		6.875954
0.5			6.202543
0.7		0.3	5.529132
	0.2		7.212660
	0.4		7.103953
	0.6		7.212660
	0.8		6.907253
0.2		0.1	6.890103
	0.2	0.2	7.056441
		0.3	7.212660
		0.4	7.359953

## Multiple linear regression

A statistical approach known as multiple linear regression examines the interdependencies between multiple explanatory variables and a single response variable. This technique examines the interconnections between various factors and key physical parameters, such as $${C}_{f}{Re}^{\raisebox{1ex}{$1$}\!\left/ \!\raisebox{-1ex}{$2$}\right.}$$(skin friction coefficient) and $${\mathrm{NuRe}}^{\raisebox{1ex}{$-1$}\!\left/ \!\raisebox{-1ex}{$2$}\right.}$$ (Nusselt number), providing valuable insights into their relationships and influences. In this study, Multiple Linear Regression (MLR) is used only as a supporting tool to complement the numerical results obtained through the Keller-box method. It is a way to create simple predictive formulas for the skin friction coefficient and Nusselt number from the numerical data. The MLR model helps estimate these quantities quickly without solving the full boundary value problem again.

This procedure can be mathematically expressed as follows [41–44]:

38 $$y=m+{m}_{1}{x}_{1}+{m}_{2}{x}_{2}+{m}_{3}{x}_{3}\dots \dots \dots \dots +{m}_{n}{x}_{n}$$

The general forms of the estimated models are as follows:

39 $${Cf}_{est}={{h}_{0}+h}_{1}{M}_{g}+{h}_{2}{A}_{P}+{h}_{3}{S}_{p}$$

40 $${Nu}_{est}={{p}_{0}+p}_{1}{E}_{C}+{p}_{2}{A}_{P}+{p}_{3}{M}_{g}$$

The solution of the regression analysis is represented by coefficients ${h}_{0},{h}_{1},{h}_{2},{h}_{3},{p}_{1},{p}_{2},{p}_{3}, and \space {p}_{0}.$  

To estimate the skin friction coefficient, a regression analysis was performed . Similarly, the heat transfer rate was modeled using a separate regression analysis . We computed both regression models using Excel. The results of the regression analysis are presented in the following models:

41 $${Cf}_{est}=-1.131243-0.327946{M}_{g}-0.270979{A}_{P}-1.333587{S}_{P}$$

42 $${Nu}_{est}=7.516168-3.360973{E}_{C}+1.565654{A}_{P}-0.5158763{M}_{g}$$

### Sensitivity analysis

The signs of the computed regression coefficients provide valuable insights into the relationships between the parameters and the respective outcomes. In standard boundary layer flows, both the Magnetic Parameter (*M*_*g*_) and the Unsteadiness Parameter (*A*_*p*_) both factors reduce drag. This suggests a unique, complex, and potentially advantageous interaction specific to this radial/hybrid/unsteady regime. The *S*_*p*_ parameter has the largest negative coefficient, confirming it as the most powerful drag-reducing mechanism in the system. The sheer magnitude (over 4 times that of *M*_*g*_) indicates that (*S*_*p*_) efficiently rearranges the momentum boundary layer to significantly lower shear stress at the wall.

The massive negative coefficient for *E*_*C*_ confirms that viscous dissipation is the dominant inhibitor of cooling. Any system modification that increases fluid friction and, consequently, dissipation (such as increasing viscosity or velocity gradients), will severely compromise Nusselt number. The large positive coefficient for *A*_*p*_ indicates that unsteadiness is the primary driver of enhanced cooling among these three terms. Running the process in an accelerated or time-dependent manner is highly beneficial for *Nu*. The negative coefficient for *M*_*g*_ confirms the adverse effect of Ohmic heating. While smaller than the *E*_*C*_ effect, it must be considered for flow control.

**Fig. 12:**
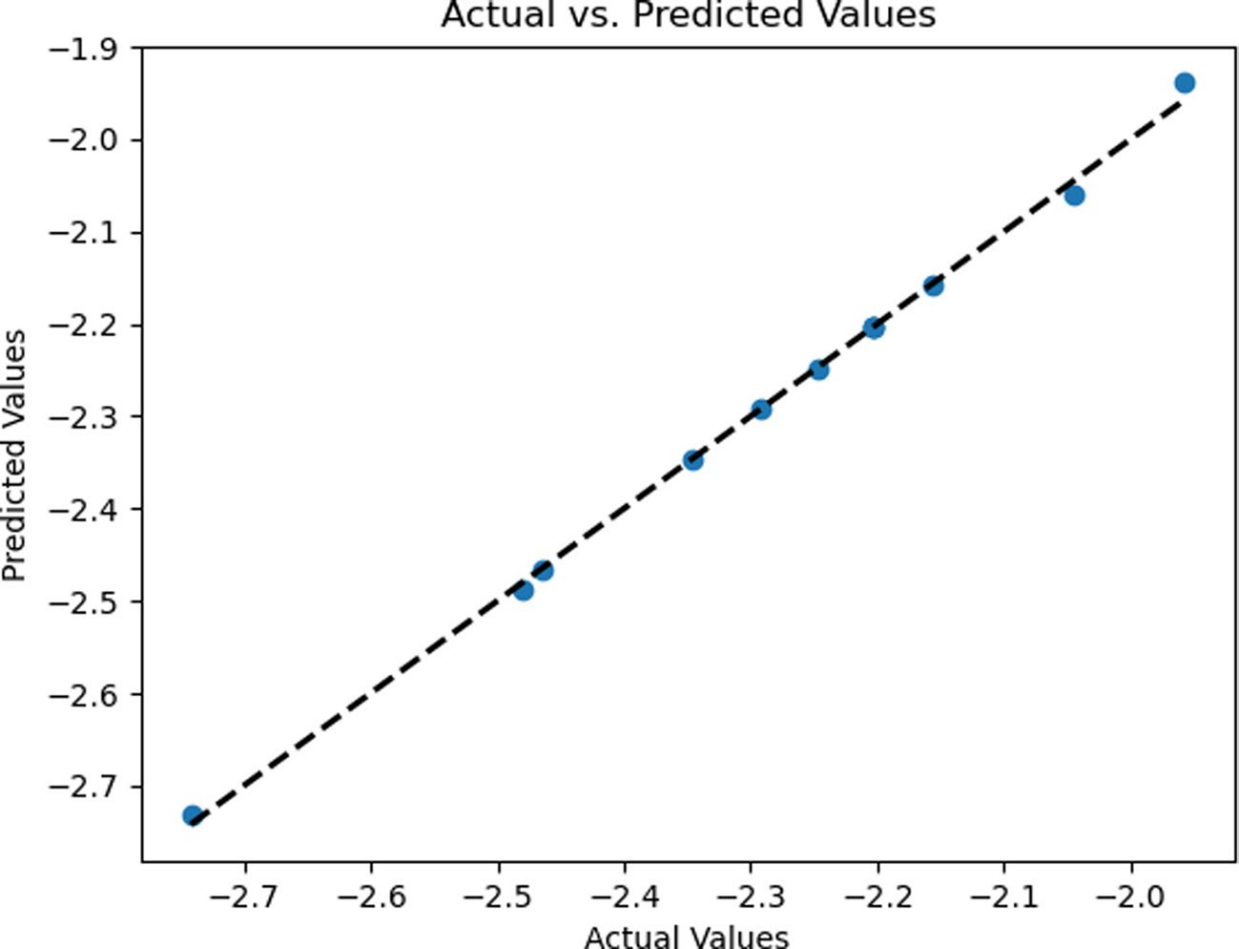
Predicted versus actual skin friction coefficients

**Fig. 13:**
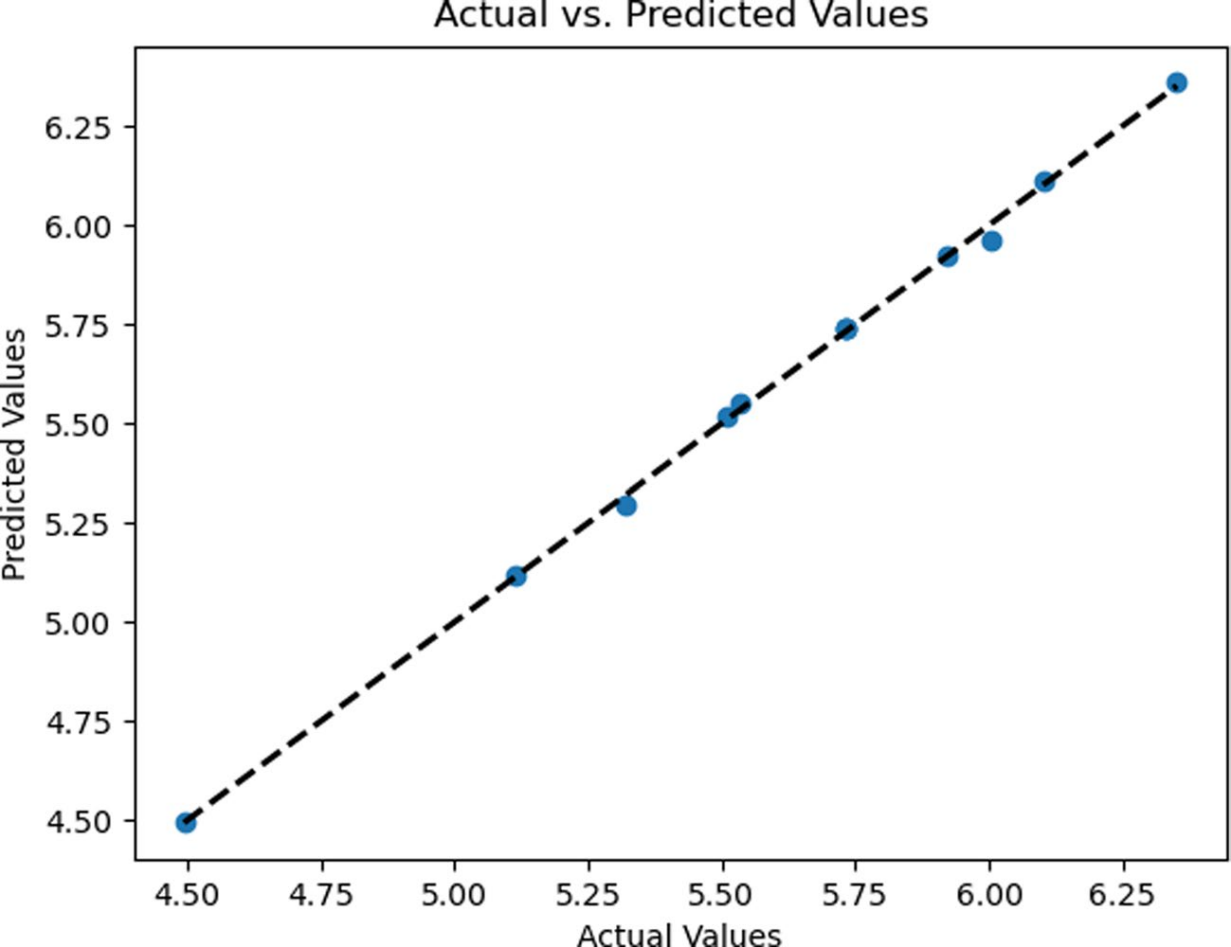
Predicted versus actual Nusselt number

The data in Tables 3 and 4 serves as evidence to support the obtained outcomes. Tables 5 and 6 contain the regression analysis results for $${C}_{f}{Re}^{\raisebox{1ex}{$1$}\!\left/ \!\raisebox{-1ex}{$2$}\right.}$$ and $${\mathrm{NuRe}}^{\raisebox{1ex}{$-1$}\!\left/ \!\raisebox{-1ex}{$2$}\right.}$$_,_ offering a concise overview of the statistical findings. An R-squared value of 0.99 indicates a strong model fit. Figures 12 and 13 illustrate the strong alignment between the numerical data points (actual values) and predicted values from the regression model, demonstrating the model's remarkable accuracy and reliability.

**Table 5:** Skin friction coefficient regression analysis

Regression outputs	
Multiple R	0.999248081
Standard error	0.009552986
R square	0.998496728
Adjusted R square	0.997933001

**Table 6:** Nusselt number regression analysis

Regression outputs	
Multiple R	0.999360722
Standard error	0.020694401
R square	0.998721852
Adjusted R square	0.998242547

## Conclusion

The present study examined the combined effects of suction, Joule heating, and viscous dissipation on the electrically conducting hybrid nanofluid (CuO–Al_2_O_3_/H_2_O) flowing over an axisymmetric stretching surface. By applying a suitable similarity transformation, the governing partial differential equations were reduced to a system of ordinary differential equations, which were subsequently solved numerically using the Keller box method in MATLAB. In addition, a statistical evaluation of the influence of various parameters on key physical responses was conducted through Multiple Linear Regression (MLR).

The findings can be summarized as follows:

- Increasing either the magnetic field strength or the suction parameter leads to a reduction in the velocity distribution, underscoring their strong influence on the fluid motion.
- The temperature profile decreases with higher Eckert numbers and magnetic field strengths, whereas the unsteadiness parameter induces an opposite trend.
- A predictive regression model for the skin friction coefficient was established as:
  $${Cf}_{est}=-1.131243-0.327946{M}_{g}-0.270979{A}_{P}-1.333587{S}_{P}$$
- A regression-based predictive expression for the Nusselt number was derived as:

$${Nu}_{est}=7.516168-3.360973{E}_{C}+1.565654{A}_{P}-0.5158763{M}_{g}$$

- The skin friction coefficient shows an inverse relationship with both magnetic field strength and suction parameters.
- The unsteadiness parameter enhances the heat transfer rate, while the Eckert number and magnetic field strength exhibit negative correlations with it.

Overall, this investigation offers meaningful insights into improving thermal energy transport in engineering applications, such as optimizing thermal management in electronic cooling systems and enhancing microfluidic heat dissipation in compact devices.

### Future scope and limitations

This works assumes the nanoparticles as spherical in shape, if not again this would be a challenge and important limitation of the problem. The analysis employs a single-phase model with simplified boundary conditions, and the results are limited to the specified parameter ranges without accounting for nanoparticle agglomeration or experimental validation. Future work may focus on experimentally validating the numerical findings and assessing the long-term stability of the nanofluid. Additional physical effects such as thermal radiation, chemical reactions, and variable fluid properties can also be incorporated to better reflect practical applications.

## Data Availability

All data used in this study are available within the manuscript.

## List of symbols

h: Initial thermal convection
$$\gamma $$: Stretching velocity
a: Positive constant
$$\sigma $$: Electrical conductivity
$$\mu $$: Viscosity coefficient
w, u: Velocity in the z and r directions
T_w_: Surface temperature
U_w_: Surface velocity
$$\rho $$: Fluid density
$${T}_{\infty }$$: Ambient temperature
C_p_: Specific heat at constant pressure
k: Thermal conductivity
$${f}^{{\prime}}{(\eta )}$$: Dimensionless velocity
B_0_: Magnetic field strength
C_f_: Skin friction coefficient
M_g_: Magnetic parameter
E_C_: Eckert number
Nu: Nusselt number
S_P_: Suction parameter
A_P_: Unsteadiness parameter
Re: Reynolds number
$$\theta (\eta )$$: Dimensionless temperature
Pr: Prandtl number
t: Time
